# Ubiquitination and the Proteasome as Drug Targets in Trypanosomatid Diseases

**DOI:** 10.3389/fchem.2020.630888

**Published:** 2021-01-28

**Authors:** Marie-José Bijlmakers

**Affiliations:** Other, Cambridge, United Kingdom

**Keywords:** trypanosoma, leishmania, ubiqutination, proteasome, drug target

## Abstract

The eukaryotic pathogens *Trypanosoma brucei*, *Trypanosoma cruzi* and *Leishmania* are responsible for debilitating diseases that affect millions of people worldwide. The numbers of drugs available to treat these diseases, Human African Trypanosomiasis, Chagas' disease and Leishmaniasis are very limited and existing treatments have substantial shortcomings in delivery method, efficacy and safety. The identification and validation of novel drug targets opens up new opportunities for the discovery of therapeutic drugs with better efficacy and safety profiles. Here, the potential of targeting the ubiquitin-proteasome system in these parasites is reviewed. Ubiquitination is the posttranslational attachment of one or more ubiquitin proteins to substrates, an essential eukaryotic mechanism that regulates a wide variety of cellular processes in many different ways. The best studied of these is the delivery of ubiquitinated substrates for degradation to the proteasome, the major cellular protease. However, ubiquitination can also regulate substrates in proteasome-independent ways, and proteasomes can degrade proteins to some extent in ubiquitin-independent ways. Because of these widespread roles, both ubiquitination and proteasomal degradation are essential for the viability of eukaryotes and the proteins that mediate these processes are therefore attractive drug targets in trypanosomatids. Here, the current understanding of these processes in trypanosomatids is reviewed. Furthermore, significant recent progress in the development of trypanosomatid-selective proteasome inhibitors that cure mouse models of trypanosomatid infections is presented. In addition, the targeting of the key enzyme in ubiquitination, the ubiquitin E1 UBA1, is discussed as an alternative strategy. Important differences between human and trypanosomatid UBA1s in susceptibility to inhibitors predicts that the selective targeting of these enzymes in trypanosomatids may also be feasible. Finally, it is proposed that activating enzymes of the ubiquitin-like proteins SUMO and NEDD8 may represent drug targets in these trypanosomatids as well.

## Introduction

*Trypanosoma brucei*, *Trypanosoma cruzi* and *Leishmania sp* are the major disease-causing trypanosomatids in humans that continue to pose a major risk to the health of millions of people worldwide, especially among the poorest in rural regions of tropical countries. These eukaryotic parasites are characterized by a single flagellum and a kinetoplast, a DNA containing region that is part of a single mitochondrion (Stuart et al., 2008). They all have an invertebrate and vertebrate host, but there are differences in their distribution, transmission and life cycle. *T. brucei*, the causative agent of Human African Trypanosomiasis (HAT), is transmitted by the tsetse fly and only found in Africa. Two different species, *T. brucei gambiense* and *T. brucei rhodesiense*, cause a slow (98% of cases) and rapidly progressing form of HAT, respectively, both of which are lethal if not treated. The *T. brucei* parasites live exclusively extracellularly in the host, during the early mild stage of the disease in the blood, lymph and interstitial spaces, and during the second stage in the central nervous system leading to severe neurological symptoms. *T. cruzi* is transmitted by blood feeding triatominae and endemic in Latin America. This trypanosomatid alternates between non-replicating trypomastigotes that circulate in the blood and dividing amastigotes, a form with only a very short flagellum, in the cytoplasm of mammalian cells after escape from a lysosomal compartment. Whereas the initial symptoms of a *T. cruzi* infection are often mild and undetected, a lifelong infection is established that in 30% of cases leads to a chronic phase of disease with severe damage to the heart and digestive system. Leishmaniasis is caused by more than twenty species of *Leishmania* that are found widespread throughout the world with the majority of disease cases occurring in Asia, Africa, and Latin America. Leishmania parasites are transmitted by the bite of a sandfly that injects the promastigote metacyclic form, which after being taken up by phagocytic cells, transforms into amastigotes that replicate inside the phagolysosome. Leishmaniasis consists of a spectrum of human diseases with symptoms varying from self-limiting ulcers to the destruction of mucocutaneous surfaces, to the deadly visceral leishmaniasis.

With the exception of *T. brucei gambiense* that mainly infects humans, these pathogenic trypanosomatids have animal reservoirs that include small and domestic animals, which makes the control of their transmission very difficult. Early diagnosis and effective medication are therefore of the utmost importance, but only a handful of drugs is available, most of which have been in use for many decades and have severe shortcomings in delivery, toxicity and efficacy (Bilbe, 2015; Rao et al., 2018). However, the past 15 years have seen various important initiatives that have increased the visibility of these neglected tropical diseases, enabled collaborations between industry, academia and the public sector for drug development on a not-for-profit basis, and increased the availability of research funding. This has resulted in significant successes particularly in the treatment of *T. gambiense* HAT for which a safe oral treatment has now become available (Dickie et al., 2020; Neau et al., 2020). More clinical trials are ongoing, also for Chagas’ disease and Leishmaniasis treatments, but failures of clinical trials have also been reported. Given the general low success rate of drug development, its low predictability and long duration plus the added anticipation of future drug resistance, multiple parallel efforts in research, drug discovery and development are required to generate a considerable pipeline of anti-trypanosomiasis drug candidates.

New combinations of existing medication as well as the optimization of dosing regimes have already resulted in rapid improvements in treatments. Furthermore, the revisiting of compounds with proven anti-trypanosome activity, research into which was abandoned due to a lack of commercial interest, has resulted in the approval and testing of new drugs (Neau et al., 2020). However, advances in analysis and screening techniques, a better knowledge of genomes, proteomes and biological pathways have also opened up many new opportunities that are currently being explored. One approach is to use phenotypic screens as a starting point for the identification of compounds with anti-trypanosomal activity. Alternatively, a target-based approach can be used in which targets are selected based on being indispensable for parasite survival, absent in humans, or sufficiently different from human orthologues, and amenable to enzymatic inhibition. Targets can then be used in high-throughput screens with compound libraries, or existing knowledge on the structure and inhibition of mammalian orthologues can be used for the design of parasite-selective inhibitors to speed up the process of drug discovery. In this review, the ubiquitin-proteasome system will be discussed as a promising source of drug targets for trypanosomal diseases. Both the proteasome and the ubiquitination machinery, which function together as well as separately, are essential for the survival of eukaryotes including trypanosomatids. Inhibitors against both processes have been identified and selective inhibition without affecting mammalian counterparts has been demonstrated for the trypanosomal proteasome and strongly suggested for the trypanosomal ubiquitin activating enzyme.

## The Ubiquitin-Proteasome System

### Ubiquitination, an Omnipresent and Versatile Protein Modification

Ubiquitination is a post-translational protein modification in eukaryotes that involves the attachment of the small protein ubiquitin to substrates which results in the degradation of substrates or in an alteration of their activity, interaction or localization (Pickart and Eddins, 2004; Komander and Rape, 2012). This reversible modification plays a crucial role in the regulation of almost every cellular process including cell cycle progression, transcriptional control, DNA repair, autophagy, protein trafficking and the removal of misfolded, damaged or old proteins.

**FIGURE 1:**
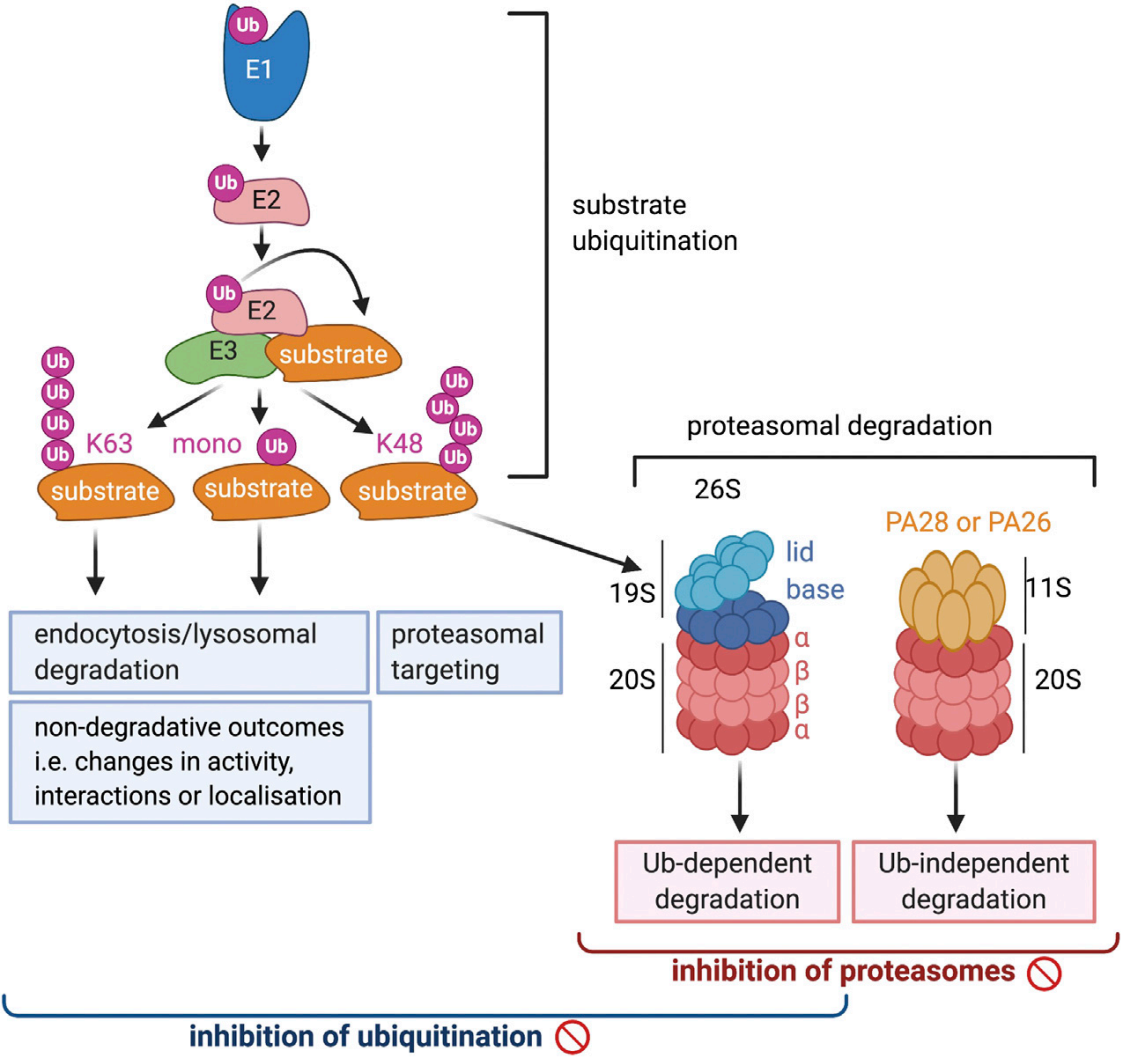
A schematic overview of the ubiquitin proteosome system. The brackets at the bottom of the diagram indicate the range of processes that will be affected by the inhibition of ubiquitination and proteosomal degradation, respectively.

The widespread and diverse roles of ubiquitination is reflected in the many different forms that this modification can take, the range of which is still expanding (Yau and Rape, 2016). Ubiquitin is primarily attached to a lysine on the substrate, but other residues such as threonine, serine and cysteine, as well as the N-terminal amino group can be modified as well. Two main types of ubiquitination are distinguished: mono-ubiquitination, when only a single ubiquitin is attached to a substrate residue, or poly-ubiquitination, when the attached ubiquitin itself undergoes sequential rounds of ubiquitination and a poly-ubiquitin chain is formed (Figure 1). Ubiquitin contains seven lysines and a free N-terminus that can all be ubiquitinated so that poly-ubiquitin chains of many different architectures can be formed, including straight Met1-linked chains as well as branched chains in which one ubiquitin molecule binds two other ubiquitins at different lysines. The most frequently occurring and best studied poly-ubiquitin chains are those linked via K48 or K63 (Swatek and Komander, 2016). K48-linked chains make up >50% of all ubiquitination and are well known to target substrates to the proteasome for degradation (Figure 1), which has now also been shown for K48/K11 branched chains and multiple mono-ubiquitination. K63-linked chains, on the other hand, are not involved in proteasomal degradation, but like single mono-ubiquitination, play a role in lysosomal targeting and degradation of plasma membrane proteins (Figure 1). Additionally, the presence of K63- and Met-linked chains, or mono-ubiquitination, can induce non-proteolytic outcomes and change the interactions, activity or localization of substrates. The roles of ubiquitin chains linked via other lysines are still emerging, but it is clear that these also contribute to degradative as well as non-degradative processes. The demonstration that attached ubiquitin can also be modified by phosphorylation and acetylation adds another level of complexity to the regulatory capacity of this modification (Herhaus and Dikic, 2015).

Ubiquitination can be reversed by de-ubiquitinating proteins (DUBs), a large and diverse group of enzymes that plays a crucial role in the regulation of ubiquitin-controlled processes (Clague et al., 2013). There is further a critical role for ubiquitin-binding domains (UBD), short protein motifs of a multitude of different signatures. These occur on many different proteins and help to translate the ubiquitin code into specific outcomes (Husnjak and Dikic, 2012).

#### Ubiquitination Enzymes in *T. brucei*, *T. cruzi* and *Leishmania*

The attachment of ubiquitin to substrates is a multistep process that requires the sequential activity of three classes of proteins (Figure 1). The first step is mediated by the ubiquitin activating enzyme (UBA1), or ubiquitin E1 (Schulman and Harper, 2009). This large multi-domain protein first adenylates ubiquitin at the C-terminus, which then reacts with a catalytic cysteine, resulting in the formation of a high energy thioester ubiquitin ∼ E1 conjugate. After binding of a second ubiquitin, the E1 can transfer the thioester bound ubiquitin to a ubiquitin conjugating enzyme or E2, where again a ubiquitin-thioester bond is formed (Stewart et al., 2016). The E2 subsequently interacts with an E3, or ubiquitin ligase, a protein that selects substrates and mediates the transfer of ubiquitin to the substrate. E3 proteins are highly diverse, characterized by an E2-binding domain that is most frequently a RING, HECT or U-box domain (Deshaies and Joazeiro, 2009). Mechanistically, these proteins can be divided into two groups: a small group that includes the HECT and RING-in-between-RING (RBR) proteins forms a covalent intermediate with ubiquitin before transferring it to substrates. A very large group that contains the majority of RING and U box proteins mediates the transfer of ubiquitin to substrates from the E2 without binding ubiquitin themselves first. E3s proteins can either function on their own or as part of large multi-protein complexes.

Going down the ubiquitination cascade, the number of proteins involved in the separate steps increases vastly (Schulman and Harper, 2009). From yeast to mammals, there is only one E1 that exclusively activates ubiquitin, UBA1 (or UBE1). In vertebrates and sea urchin UBA6, the E1 for the ubiquitin-like protein FAT10, can activate ubiquitin as well, but UBA1 is responsible for more than 90% of all ubiquitination (Pelzer et al., 2007; Liu et al., 2017). At the next level down in mammalian genomes, approximately 40 ubiquitin E2 conjugating proteins can be found. Collectively, these E2s interact with more than 600 ubiquitin E3s, which each recruit sets of substrates that number from a few to several hundreds.

Consistent with this hierarchy, UBA1 is an essential protein. This has been demonstrated by gene ablation studies in yeast and *C. elegans* where UBA1 is the only ubiquitin E1 (McGrath et al., 1991; Kulkarni and Smith, 2008), but also in various human cancer cell lines (Xu et al., 2010) where UBA6 is apparently not able to compensate for the loss of UBA1. This vital role is further illustrated by the cytotoxic effect of UBA1 inhibitors on mammalian cells (Barghout and Schimmer, 2021).

In *T. brucei*, *T. cruzi* and Leishmania, ubiquitination has not yet been extensively investigated, but it is clear from genome-wide RNAi screens, genome analysis, and reports on specific cellular processes that also in these parasites this modification plays an essential and widespread role. Genome-wide analysis shows that genes encoding for orthologues of ubiquitin E1, E2 and E3 proteins are all present in these species, as are enzymes involved in de-ubiquitination (Gupta et al., 2018). Ubiquitin E1 proteins can be identified by the presence of two ThiF/MoeB motifs and a C-terminal ubiquitin fold domain (UFD) (Schulman and Harper, 2009). Surprisingly, there are two orthologues of UBA1 in trypanosomatids, called TbUBA1a and TbUBA1b in *T. brucei* (Chung et al., 2008; Boer and Bijlmakers, 2019). Of these two, TbUBA1a is the most closely related to human UBA1 (hUBA1): it is similar in length and 36% identical at the amino acid level. TbUBA1b on the other hand, has an N-terminal extension, several insertions of multiple amino acids and is only 28% identical to hUBA1. The 2 *T. brucei* UBA1s are also only 24% identical to each other, suggesting that they may have arisen from an early gene duplication. In agreement with this, orthologues of both proteins are present in all other kinetidoplastids sequenced so far, but no TbUBA1b equivalents are found outside this taxus. *T. cruzi* and *Leishmania* have orthologues that are, respectively, 70% and 55% identical to TbUBA1a, and orthologues that are 68% and 59% identical to TbUB1b (Boer and Bijlmakers, 2019). TbUBA1b does not show any extensive similarity to the other two E1 proteins with two ThiF domains, UBA6 and UBA7.

Consistent with a function as a ubiquitin E1, TbUBA1b RNAi knockdown in the blood stream form (BSF) of *T. brucei* results in an overall reduction in ubiquitination (Chung et al., 2008). Furthermore, like TbUBA1a, TbUBA1b can activate ubiquitin *in vitro*, which was measured by the transfer of ubiquitin to an E2 (Boer and Bijlmakers, 2019). Both TbUBA1a and TbUBA1b are expressed in the procyclic insect form as well as in the blood stream form of *T. brucei* (Siegel et al., 2010) (https://tritrypdb.org/). While the significance of this expression of two different UBA1s is not yet clear, data from a genome-wide gene ablation screen show that both proteins are essential for *T. brucei* survival (https://tritrypdb.org/). In this screen, in which ∼10,000 individual genes were targeted by tetracyclin-inducible RNAi, TbUBA1b ranks among the 1% of genes with the severest growth defect upon knockdown (Alsford et al., 2011). Targeting of TbUBA1b led to an 88% reduction in viability after three days of induced RNAi, while targeting of TbUBA1a resulted in a 55% reduction at this time.

The E2 proteins of these parasites have not yet been studied, but approximately 15 E2 genes can be identified in their genomes based on the presence of a UQ-con motif (Gupta et al., 2018). E2s of *T. brucei* are between 26.5–74.3% identical to their human orthologues and based on sequence similarities functions may be attributed to some. For instance, the protein with the highest sequence identity to human E2s, Tb927.5.1000, is an orthologue of the proteins of the UBE2D (UbcH5) family, which are the most versatile E2s with roles in many processes (Brzovic and Klevit, 2006). In the whole-genome RNAi screen, the knockdown of Tb927.5.1000 decreases viability by 77% (Alsford et al., 2011), suggesting that the encoded E2 also has a widespread role in *T. brucei*. Along similar lines, *T. brucei* contains genes for orthologues of UBE2N (Ubc13) and UBE2V1, two E2s that specifically function in the formation of K63-linked poly-ubiquitin chains. The Ubc13 orthologue in *T. brucei* is 66% identical to the human protein, and its knockdown results in a 87% reduction in viability (Alsford et al., 2011), strongly suggesting that K63-linked ubiquitination plays an important role in *T. brucei*.

Based on the presence of RING, HECT or U-box domains, approximately 60 E3 ligases have been identified in the genomes of each individual trypanosomatid (Gupta et al., 2018). The group of RING domain proteins is by far the largest and shows the predicted heterogenousity in architectures as seen in other eukaryotes. Additionally, cullins and F-box proteins that constitute subunits of large E3 complexes are present. The trypanosomatid E3s have not yet been studied in any detail with the exception of a few. A cullin-RING CRL4^WDR1^ was identified in *T. brucei* that controls the levels of Polo-like kinase (TbPLK), a protein with several essential roles in cell division. The impediment of TbPLK ubiquitination by CRL4^WDR1^ was shown to severely affect its function (Hu et al., 2017). An intriguing finding is the identification of SPRING, a RING E3 ligase that is unique to *T. cruzi* (Hashimoto et al., 2010). This protein is secreted by *T. cruzi* and localizes to the nucleus of host cells where it may ubiquitinate proteins. So far SPRING has been demonstrated to have *in vitro* E3 activity, but its significance for *T. cruzi* infection has not yet been shown.

#### Processes Controlled by Ubiquitination in *T. brucei*, *T. cruzi* and *Leishmania*

The list of processes in which ubiquitination plays a role is still expanding. For a long time ubiquitination has been known to target damaged and old proteins to the proteasome and thus to be important for protein homeostasis. This is now understood to extend to newly synthesized proteins that have failed to fold properly including those that are synthesized in the ER (Wu and Rapoport, 2018). In the latter case, proteins are translocated out of the ER and ubiquitinated by an ER-associated E2/E3 couple, Ubc6 (UBE2J)/Hrd1, a process known as ER-associated degradation (ERAD). Failure of ERAD induces ER stress, which induces a response mechanism that leads to programmed cell death. A functional ERAD pathway has been shown to be operational in *T. brucei* (Field et al., 2010; Tiengwe et al., 2016; Tiengwe et al., 2018), and orthologues of Ubc6, Hrd1 and other critical components are present. Also in *T. cruzi,* the retrotranslocation of proteins out of the ER has been demonstrated (Labriola et al., 2010). Based on genome analysis it has been proposed that in these parasites a minimal ERAD pathway with little redundancy exists. The resulting higher dependence on individual proteins has been proposed to make this crucial process an attractive drug target (Harbut et al., 2012).

In addition to essential housekeeping roles, ubiquitin-dependent proteasome degradation regulates processes that require the dynamic control of protein levels. Examples of these are the degradation of specific cyclins at different stages of the cell cycle which is essential for cell cycle progression, or the degradation of proteins in cell signaling. Both genomic and functional data show that, like in other eukaryotes, ubiquitination plays an important role in cell cycle progression of trypanosomatids. The large E3 protein complex SCFC (Skp1-CUL1-F-box complex) degrades cell cycle regulators and contains among its fixed components the E2 CDC34 (UBE2R1). Orthologues of Skp1, CULLIN1, RBX1 and CDC34 are all present in *T. brucei*, and the depletion of TbCDC34 results in cell death (Rojas et al., 2017). Further evidence comes from the presence of cyclins with short half-lives, which has been shown for both *T. brucei* and *T. cruzi*. In the presence of proteasome inhibitors, the half-lives of these proteins significantly increase and poly-ubiquitinated forms accumulate (Hellemond and Mottram, 2000; Renzo et al., 2016) The Anaphase promoting complex/cyclosome, APC/C, is another large E3 complex required for cell cycle regulation. Ten APC/C subunits were identified in *T. brucei* and the RNAi knockdown of one of these, AP2, resulted in cell cycle arrest and accumulation of poly-ubiquitinated cyclin B (Bessat et al., 2013). In Leishmania, the cell cycle dependent degradation of a kinesin also suggests the involvement of the ubiquitin-proteasome system (Dubessay et al., 2006). Furthermore, in this parasite the ubiquitin mediated degradation of the enzyme methione adenosyl transferase has been reported. This degradation is believed to be important to control the levels of this metabolically important protein (Pérez-Pertejo et al., 2011).

Non-proteasome dependent outcomes of ubiquitination are also numerous and required for many essential cellular functions that includes a role in regulating the expression of cell surface transmembrane proteins. This has also been shown to be important in *T. brucei* for two abundant transmembrane proteins, ISG65 and ISG75. The mono-ubiquitination of these proteins at the C-terminal domain provides a signal for internalization and targeting to an endosomal/lysosomal compartment for proteasome-independent degradation (Chung et al., 2008; Leung et al., 2011), which occurs in both the BSF and insect form of *T. brucei*. Furthermore, the knockdown of two de-ubiquitinating proteins, TbUsp7 and TbVdu1, was demonstrated to alter the abundance not only of ISG65 and ISG75 but also of several other proteins at the plasma membrane. The ESCRT machinery, large protein complexes that are required for ubiquitination, internalization and sorting of cell surface proteins, are believed to be present in trypanosomatids (Leung et al., 2011; Silverman et al., 2013) although no orthologues of the E3 ligases known to be associated with this complex have been found.

Thus, it is clear that ubiquitination is involved in many essential processes in trypanosomatids. There is evidence for K48-linked, K63-linked and mono-ubiquitination, and for proteasome-dependent as well as proteasome-independent outcomes. The importance of ubiquitination in these parasites is most succinctly illustrated by the severe impairment of viability upon knockdown of the TbUBA1s.

### The Proteasome, the Major Cellular Protease

#### Structure and Function of the Proteasome

The 26 proteosome is a large structure in the cytoplasm and nucleus that is composed of a proteolytic 20 S core particle capped on either or both sides by a regulatory 19 S particle (Bard et al., 2018) (Figure 1). The core particle is barrel-shaped and consists of four rings of seven proteins each, the two outer rings containing *α* subunits and the two inner rings *ß* subunits. Three of the *ß* subunits, β1, β2 and β5, have proteolytic activity and cleave after negatively charged (caspase-like activity), positively charged (trypsin-like activity) or large hydrophobic amino acids (chymotrypsin-like activity), respectively. The six catalytic sites are located in the hollow of the barrel through which substrates pass resulting in their cleavage into peptides of 15–20 amino acids. All three proteolytic activities are needed for efficient protein degradation although the relative contribution of each may vary for different substrates.

The proteolytic *ß* subunits are N-terminal threonine proteases that are synthesized as proproteins and undergo autolytic cleavage during proteasome assembly which exposes their catalytic N-terminal threonine. The hydroxyl group of the threonine side chain acts as the nucleophile after its deprotonation and attacks the carbonyl carbon atom of the bond to be cleaved, forming a covalent acyl-enzyme tetrahedral intermediate and releasing the first product (Fenteany et al., 1995; Marques et al., 2009). Subsequent hydrolysis of the intermediate releases the second product and reconstitutes the active site of the protease. The deprotonation of the threonine hydroxyl group was reported to be enhanced by the free N-terminal amino group although a Lys at position 33 has more recently been shown to be involved in this (Huber et al., 2016).

One of the functions of the 19 S regulatory particle is to open the channel of the core particle which otherwise remains closed to prevent unwanted proteolytic degradation (Bard et al., 2018). The 19 S regulator is composed of two parts, a base and a lid. The base makes direct contact with the *α* subunits of the 20 S core particle and contains a ring of six Rpt subunits with ATPase activity that are required for the unfolding and translocation of substrates. This ring associates with three non-ATPase Rpn subunits, Rpn1, Rpn2 and Rpn13 that have structural (Rpn2) and ubiquitin-binding functions (Rpn1 and 13). The lid, which sits on top of the base but also makes direct contact with the core particle, is composed of another nine subunits whereas the subunit Rpn10, another ubiquitin-binding protein, cross-bridges the base and lid. The majority of subunits of the lid have structural functions, while Rpn11 is a de-ubiquitinase (Bard 2018 review). In addition, there are two more de-ubiquitinating enzymes stably associated with the proteasome, Usp14 and Uch37. Apart from the ubiquitin-binding subunits of the 19S, cytoplasmic shuttle factors play a role in delivering poly-ubiquitinated substrates to the proteasome.

The majority of proteins that are degraded by the proteasome are ubiquitinated, but ubiquitin-independent degradation takes place as well (Vigneron and Eynde, 2014). Examples include the degradation of proteins with intrinsically disordered regions and proteins damaged by oxidation. Such degradation can involve the 20 S core particle alone, or the 20 S particle associated with an alternative regulatory particle, 11 S or PA28. PA28 is a heptameric ring structure without ATPase activity that stimulates the activity of the 20 S core particle by opening the gate occupied by the loops of the *α* subunits (Figure 1) (Rechsteiner and Hill, 2005). The mammalian 11 S regulator can occur in two different forms either composed of a mix of PA28α and PA28β subunits, or composed of a single subunit, PA28γ. PA28αβ is induced by interferon-γ and plays a role in antigen presentation by MHC I proteins in the adaptive immune response, PA28γ is not induced by IFN-γ and is exclusively found in the nucleus. The 20 S associated with an 11 S regulator does not function in general proteolysis but rather has specific functions such as the degradation of peptides, disordered or oxidized proteins.

The proteasome is responsible for 80–90% of protein degradation and is thus the most important protease in the cell. The essential role of the proteasome is clear from the cytotoxic effect of proteasome inhibitors on eukaryotic cells.

#### Proteasomes of Trypanosomatids

The proteasomes of trypanosomatids resemble those of other eukaryotes but distinguishing features have been reported as well (Muñoz et al., 2015). The most extensively studied is the proteasome of *T. brucei*. Early purification studies failed to detect an association between the 20 and 19 S particle of *T. brucei* (Shao-bing et al., 1996) but this was found to result from an unusual instability of the 26 S proteasome during cell lysis (Li et al., 2002). In this respect, the *T. brucei* 26 S proteasome differs from that of other species including human, but also *T. cruzi* (Diego et al., 2001) and *Leishmania* (Robertson, 1999).

Another surprising observation was that a large fraction of the *T. brucei* 20 S core particle was isolated in a highly active form (To and Wang, 1997). This is the result of an association with an 11 S regulator, PA26, which is a heptameric ring of a single subunit without ATPase activity that associates with the *α* subunits of the 20 S particle, thereby opening its gate (Yao et al., 1999; Whitby et al., 2000). Whereas PA26 resembles in structure and activity PA28, it is exclusively expressed in trypanosomatids and does not have extensive sequence similarity to PA28 subunits (Yao et al., 1999). PA26 can activate the 20 S proteasomes of mammals, but vice versa, mammalian PA28 can not activate the *T. brucei* 20 S proteosome (Yao et al., 1999). This again suggests that there are structural differences between the *T. brucei* and mammalian proteasomes. Like PA28, PA26 is involved in ubiquitin-independent degradation of peptides rather than proteins (Yao et al., 1999; Hill et al., 2002), but its physiological role is not yet clear. Both PA26 and the 19 S regulator are present in *T. cruzi* and locate to the cytoplasm, nucleus and kinetoplastid (Cardoso et al., 2011). The exposure of *T. cruzi* to gamma irradiation leads to an upregulation of PA26, suggesting that it may be important under stress conditions (Cerqueira et al., 2017). However, despite its abundance in *T. brucei*, ablation of PA26 did not affect the viability of the procyclic insect form of this parasite (Li et al., 2002).

The composition of the 19 and 20 S particles of trypanosomatids is the same as in other eukaryotes (Huang et al., 2001; Li et al., 2002; Wang et al., 2003; Muñoz et al., 2015). Thus, the 20 S core particle contains seven distinct *α* and seven distinct *ß* subunits with three catalytic *ß* subunits, but there are no equivalents of the additional three IFN-γ inducible *ß* subunits that are found in vertebrates. The overall sequence identity of the *T. brucei α* subunits with human orthologues ranges between 37–54% and for *ß* subunits between 33–50%. The depletion of either individual *α* or *ß* subunit by RNAi in *T. brucei* leads to an accumulation of ubiquitinated proteins and a lack of cell growth (Li et al., 2002). Among the 19 S subunits, the six Rpt ATPases show the highest level of sequence conservation with 54–69% identity to human counterparts, whereas the Rpn subunits are between 20–46% identical. The loss of expression of all individual Rpt and Rpn proteins by RNAi results in an increase in ubiquitinated proteins, cell cycle arrest and subsequent cell death (Li et al., 2002; Li and Wang, 2002). This illustrates well the importance of ubiquitin-dependent proteasome degradation for the survival of *T. brucei*.

The proteolytic activity of the *T. brucei* 20 S proteasome displays several unique features. Initial experiments reported an unusual high trypsin-like activity, in contrast to the dominant chymotrypsin-like activity of mammalian proteasomes (Shao-bing et al., 1996). This was based on differences in relative activities of *T. brucei* and rat proteasomes against a limited set of peptides with different amino acids at P1, the site of amide bond lysis. An analysis with large peptide libraries in which also the amino acid requirements at the P2, P3 and P4 positions were taken into account, showed that *T. brucei* proteasomes preferably cleave after hydrophobic residues and therefore also have a dominant chymotrypsin-like activity (Wang et al., 2003). Nevertheless, differences between *T. brucei* and human proteasomes were also obvious in these experiments. Whereas both proteasomes prefer hydrophobic residues at the P2, P3 and P4 positions, only human proteasomes can recognize peptides with His, Lys, Asp and Glu at these locations. In contrast, only the *T. brucei* 20 S proteasome efficiently cleaved peptides with Gln at P1 (Wang et al., 2003). Overall it was concluded from the analysis of the peptide library experiments that the substrate specificity of the *T. brucei* β5 subunit is very broad while that of β2 is very limited.

Other differences between human and *T. brucei* proteasomes were observed in labeling experiments with the general probe ^125^I-TyrLeu3-VS, a peptide vinylsulfone, that irreversibly binds to the active site of all three mammalian proteolytic β subunits (Bogyo et al., 1998). Strikingly, the *T. brucei* β1 subunit does not react with this probe (Wang et al., 2003), and a similar result was found with a related fluorescently labeled probe, Me4BodipyFL-Ahx3Leu3VS (Wyllie et al., 2019). Furthermore, there was an absence of cleavage at acidic residues in the peptide library experiments (Wang et al., 2003). In the presence of PA26, when a drastic broadening of substrate selectivity was seen and also cleavage at acidic amino acids occurred, labeling of β1 with the ^125^I-TyrLeu3-VS probe was still not detected. This suggests that the β1 subunit of *T. brucei* may be inactive or have unknown substrate selectivity. Further differences in substrate selectivity of the β2 and β5 subunits with the human proteins were also observed. The vinylsulfone ^125^I-NP-L2N-VS, labels exclusively the β2 subunit of *T. brucei*, but both β2 and β5 of human origin (Wang et al., 2003). Furthermore, the peptide suc-Leu-Leu-Val-Tyr-AMC is cleaved exclusively by β5 in human proteasomes, but by β5 and β1 or β2 in the *T. brucei* proteasome (Zmuda et al., 2019).

The proteasome activity of *T. cruzi* has not been as extensively studied, but for this parasite all three proteolytic activities were readily detected, including the caspases-like activity assigned to β1 (González et al., 1996). For *Leishmania*, where the analysis is even more preliminary, differences between two different species were detected. The proteasomes of *L. chagasi* showed a three times higher trypsin-than chymotrypsin-like activity (Chagasi) (Silva-Jardim et al., 2004), but this was not the case for *L. mexicana* (Robertson, 1999). The activity of β1 was not studied for either. More detailed analyses are required to map the substrate selectivity of the *T. cruzi* and *Leishmania* proteasomes.

## Inhibition of Ubiquitination

### UBA1 Inhibitors

An overall inhibition of ubiquitination disrupts proteasomal degradation, lysosomal degradation as well as non-degradative ubiquitin-regulated processes (Figure 1), and thus affects a wide range of vital eukaryotic processes.

UBA1 is by far the preferred drug target of the ubiquitinating enzymes because of its position at the apex of the ubiquitination cascade (Barghout and Schimmer, 2021). Moreover, UBA1 has two catalytic domains, of which the ATP-binding adenylation domain is amenable to inhibition by nucleotide mimetics, a well explored strategy of inhibition. The catalytic cysteine domain can be targeted as well, but thiol reactive molecules often suffer from a general high reactivity and therefore a limited selectivity. A similar problem exists for the targeting of E2 proteins that also have an active site cysteine. Finally, the possibility of generally targeting E3 proteins is limited because these proteins are highly diverse and the majority do not possess catalytic activity. Instead, they function by optimally placing the E2-bound ubiquitin for attack by lysines of the substrate. Nevertheless, very few UBA1 inhibitors have been developed so far, but this is changing because of the success of proteasome inhibitors in the clinic. The inhibition of ubiquitination is now seen as a possible alternative or complementary approach to proteasome inhibition in cancer treatment.

**FIGURE 2:**
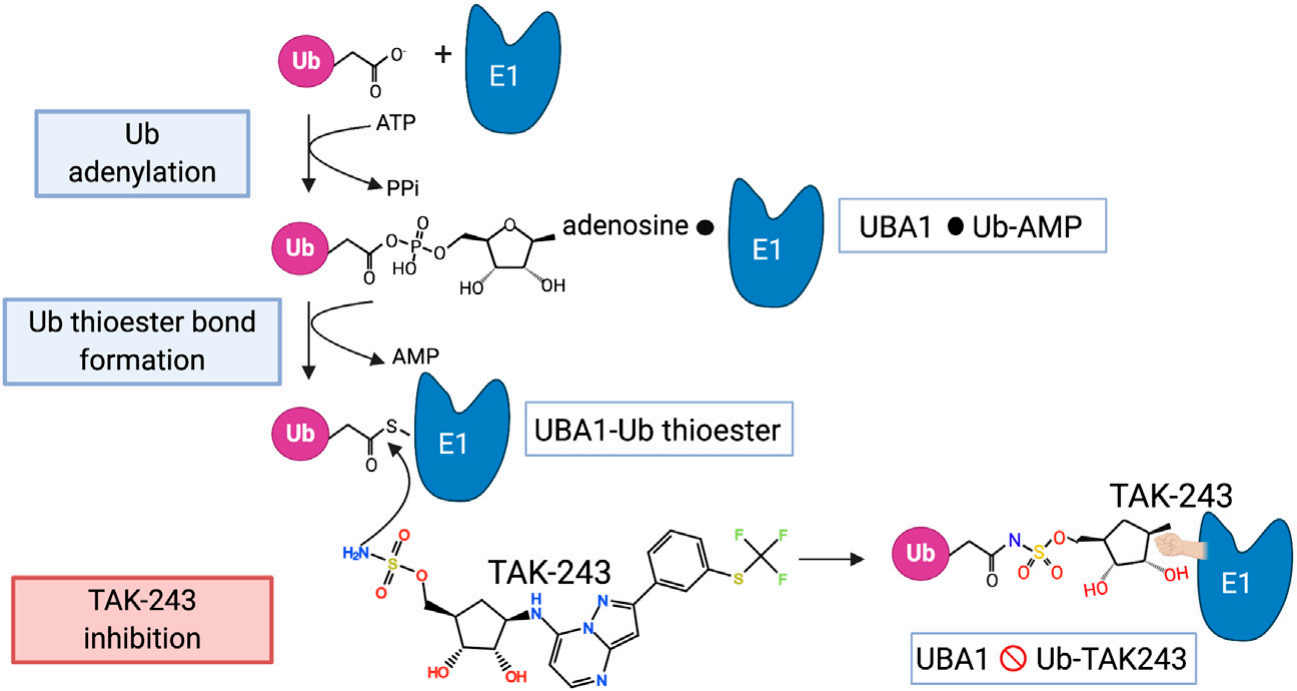
The mechanism of UBA1 inhibition by TAK-243. The steps involved in the activation of ubiquitin by UBA1 are shown as well as the mode of action of TAK-243 as described in the main text. The black circle indicates a non-covalent association.

The activation of ubiquitin by UBA1 starts with the adenylation of the C-terminus of ubiquitin in the presence of ATP, thus forming a ubiquitin∼AMP adduct and releasing PPi (Schulman and Harper, 2009) (Figure 2). This takes place at the adenylation domain (AAD) and is followed by a conformational change that brings the catalytic cysteine domain (CCD) closer, so that a UBA∼ubiquitin thioester bond is formed and AMP is released. After adenylation of a second ubiquitin, the thioester-bound ubiquitin is primed for transfer to an E2 via a transthiolation reaction. The E2s are recruited to UBA1 by its UFD.

The first indication that UBA1 inhibition can be achieved, came from the synthesis of an ubiquitin-conjugate, adenosyl-phospho-ubiquitinol (APU), that mimics the ubiquitin-adenyl intermediate but is not hydrolyzable and inhibits UBA1 in an ATP-competitive manner (Wilkinson et al., 1990). Although this inhibitor is very effective in cell free assays it is unable to enter cells. The same is true for two other ubiquitin conjugate inhibitors Ub-AMSN and Ub-AVSN (Lu et al., 2010).

The first cell-permeable inhibitor is the small molecule PYR-41, which was identified in a screen for E3 inhibitors but showed a more potent effect against UBA1 (Yang et al., 2007). The furan ring of this molecule is essential for its inhibitory activity, but the precise binding of PYR-41 to UBA1 has not yet been established. This compound inhibits the formation of the ubiquitin-thioester bond without affecting the ubiquitin-adenylation step (Ungermannova et al., 2011). When added to cells, PYR-41 inhibits both proteasome-dependent degradation of cyclins and lysosomal degradation of EGFR, as expected from an UBA1 inhibitor (Yang et al., 2007). Transformed cells were found to be more susceptible to killing by PYR-41 than non-transformed cells. However, this molecule also induces covalent protein cross-linking in cells and is therefore not a selective UBA1 inhibitor (Kapuria et al., 2011). The molecule PYZD4409 is structurally related to PYR-41 and was found in a small-scale screen for UBA1 inhibitors focused on pyrazolidines (Xu et al., 2010). In cells, this molecule inhibits the degradation of short-lived molecules such as p53 and cyclin D3, but it is expected to display the same off-target effects as PYR-41.

Two molecules, the natural product largazole and NSC624206 were isolated in cell-based screens for inhibitors of the degradation of the cell cycle inhibitor p27 (Ungermannova et al., 2011; Ungermannova et al., 2012). Largazole inhibits UBA1 at the adenylation step *in vitro* but its cellular toxicity may primarily be attributed to HDAC inhibition (Ying et al., 2008; Barghout and Schimmer, 2021). NSC624206 inhibits UBA1 at the thioester formation step (Ungermannova et al., 2011). NSC624206 was co-crystallized with *S. pombe* UBA1, but its orientation in this structure was not related to its inhibitory activity so its mechanism is not yet clear (Lv et al., 2017).

**FIGURE 3:**
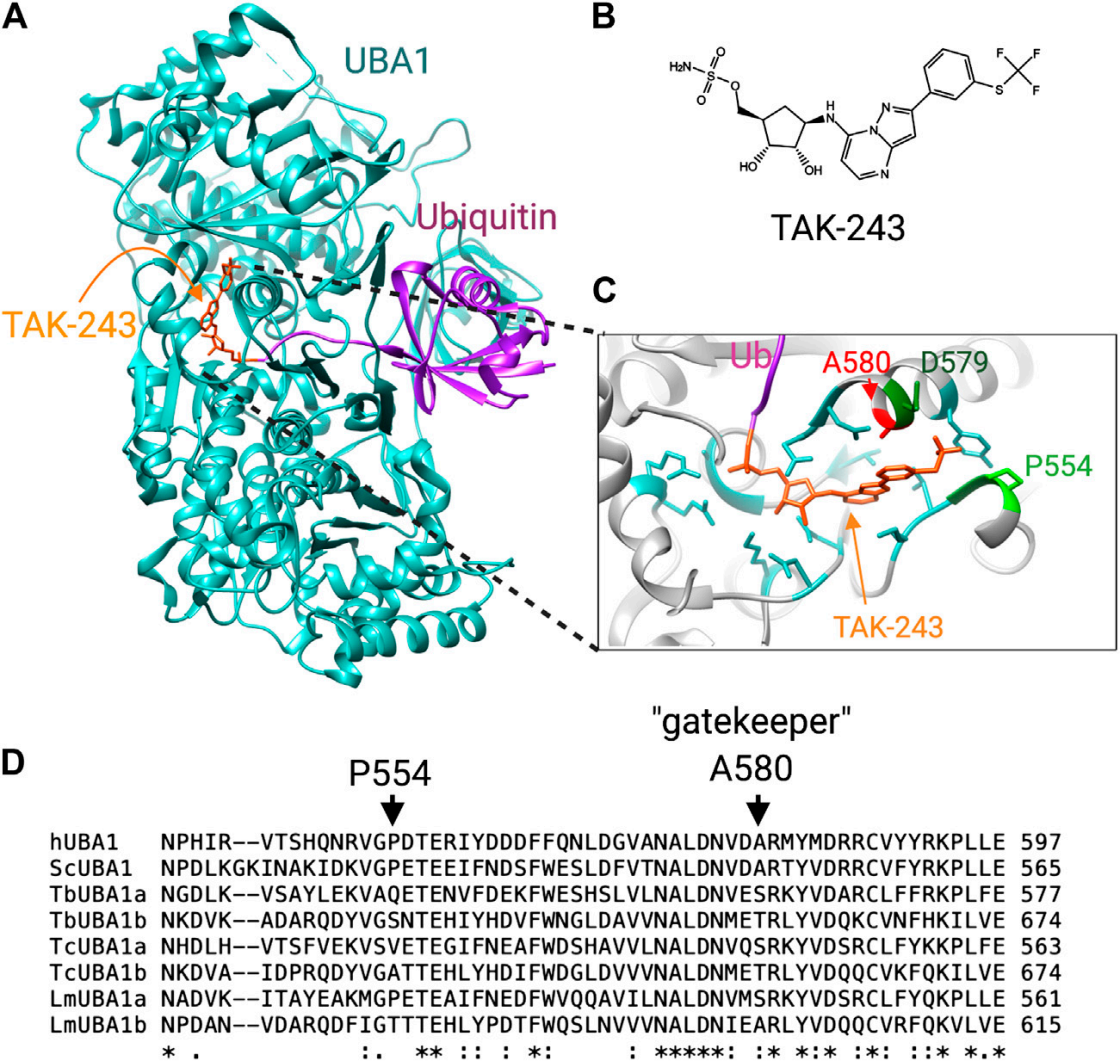
Differences at the TAK-243 binding site between human and trypanosomatid UBA1s. **(A)** The crystal structure of *S. cerevisiae* UBA1 complexed with a TAK-243-ubiquitin adduct (PDB: 5L6J). **(B)** chemical structure of TAK-243. **(C)** Detailed view of the TAK-243 binding site in which the crucial differences with the *T. brucei* TbUBA1s are labeled. **(D)** Amino acid sequence comparison of human, yeast and trypanosomatid UBA1s. The residues that contribute to the resistance of TbUBA1a and TbUBA1b to TAK-243 are indicated with arrows.

A great advance in UBA1 targeting came with the development of TAK-243 (previously called MLN7243), a potent selective UBA1 inhibitor with a well-established mode of action (Hyer et al., 2018) (Figure 2). TAK-243 belongs to the same class of molecules as MLN4924 (pevonedistat) that selectively inhibits UBA3 (Soucy et al., 2009), the E1 for the ubiquitin-like protein Nedd8. MLN4924 is currently tested in phase 1 and 2 clinical trials of various malignancies because of the importance of cullin E3 neddylation in cell cycle progression. TAK-243 and MLN4924 are adenosyl sulfamates that act as AMP mimetics and inhibit E1s in a unique manner that has been termed substrate-assisted inhibition since it requires the activity of the E1 in adenylation and thioester bond formation of ubiquitin (or Nedd8) (Brownell et al., 2010; Barghout and Schimmer, 2021). Thus, the mechanism of TAK-243 involves its binding to the ATP binding site of UBA1, from where its sulfamate NH_2_ attacks the UBA1∼ubiquitin thioester bond. The result is a stable TAK243∼ubiquitin adduct that cannot be released from the enzyme and inhibits all further ubiquitin activation (Hyer et al., 2018) (Figure 2). In crystal structures TAK-243 can be seen to occupy the site normally taken by AMP where it is bound to ubiquitin (Misra et al., 2017) (Figure 3). TAK-243 inhibits cell free UBA1 with an IC50 that is respectively 6, 28 and 850x lower than that for UBA6, UBA3 and UBA2 (SUMO E1), and >5,000 × lower than that for UBA7 (ISG15 E1) and ATG7 (E1 involved in autophagy) (Hyer et al., 2018). In agreement with UBA1 as the main target, TAK243 inhibits the degradation of short-lived molecules in cells, induces ER stress and alters DNA repair. It also arrests cells at the G1 and G2/M phase of the cell cycle and kills a wide variety of tumor cells at EC50s between 0.006 and 1.31 µM. TAK-243 inhibits the proliferation of xenograft tumors in mice and the presence of TAK-243∼ubiquitin adducts can be detected in tumor tissues (Hyer et al., 2018).

### Targeting Trypanosomatid UBA1s

The inhibition of *T. brucei* UBA1s by TAK-243 has been investigated by *in vitro* transthiolation assays and showed that both UBA1s are virtually resistant to TAK-243 (Boer and Bijlmakers, 2019). The inhibition of TbUBA1a required >500-fold higher concentrations than that of hUBA1, and the inhibition of TbUBA1b required >100-fold higher concentrations. The explanation for this resistance comes from important differences with the human protein at the TAK-243 binding pocket. This shows promise for the design of inhibitors with the opposite property of inhibiting the parasite, but not human UBA1.

The mode of TAK-243 binding has been demonstrated in crystal structures of humanized yeast UBA1 with bound TAK-243-ubiquitin. This revealed that this molecule protrudes further into the AAD domain than AMP (Misra et al., 2017; Hyer et al., 2018). Specifically, the molecule extends into a pocket where the trifluoromethyl-thiophenyl group adopts the shape of a hook (Misra et al., 2017). The resistance of the TbUBA1s to TAK-243 was found to stem from amino acid differences with hUBA1 at this pocket: of the four residues that make tight contacts with the trifluoromethyl-thiophenyl in the humanized yeast UBA1, three are different in both TbUBA1a and TBUBA1b (Boer and Bijlmakers, 2019). The most striking difference is that of the gatekeeper residue, A580 in hUBA1 (Figure 3). This alanine is highly conserved throughout evolution and is found at equivalent positions in other E1s as well. This residue plays a key role in the binding of adenosyl sulfamate inhibitors, which was first demonstrated for UBA3 by the induction of MLN4924 resistance in tumor cells and xenografts (Milhollen et al., 2012; Toth et al., 2012). In two thirds of MLN4924 resistant cells, the UBA3 equivalent of the gatekeeper, A171, was replaced by a bulkier amino acid, such as a threonine or aspartic acid.

In UBA1, a similar role for this residue was demonstrated by the reduced sensitivity of a A580T mutant to inhibition by TAK-243 (Misra et al., 2017). Moreover, an A580S mutation was detected in an AML cell line with evolved resistance to TAK243 (Barghout et al., 2019). However, despite the high evolutionary conservation of this alanine, TbUBA1a contains a serine at the equivalent position (S560) and TbUBA1b a threonine (T657) (Boer and Bijlmakers, 2019) (Figure 3). Consistent with an important role in TAK243 resistance, the substitution of these residues to an alanine significantly increased sensitivity to TAK243. This was particularly striking for TbUBA1b where an T657A mutation increased sensitivity to TAK243 ∼100 times. The second important difference at the “trifluoromethyl-thiophenyl” pocket is the presence of a glutamine (Q534) in TbUBA1a and a serine (S631) in TbUBA1b at the equivalent position of P554 in hUBA1. Substitution of these residues by a proline further increased the sensitivity to TAK-243 of the TbUBA1s, with the greatest effect on TbUBA1a. The difference at the third residue at this pocket, which is an aspartic acid in hUBA1 (D579) and a glutamic acid in TbUBA1a and TbUBA1b, contributes little to TAK-243 resistance consistent with its more conserved nature. Overall, the humanizing mutations increased TAK-243 sensitivity of TbUBA1a ∼25x and that of TbUBA1b > 100x, illustrating the importance of these residues in the distinct susceptibility to TAK-243 inhibition of the *T. brucei* UBA1s compared to the human protein (Boer and Bijlmakers, 2019).

The presence of a threonine at the gatekeeper position of UBA3 has been predicted to clash with the indane group of MLN4924, based on structural modeling (Milhollen et al., 2012). Similarly, the serine and threonine at this position in TbUBA1a and TbUBA1b respectively, are predicted to clash with the thiophenyl group of TAK243 (Figure 3). Additionally, structural modeling predicts that the “trifluoromethyl-thiophenyl” pocket is considerably larger in both TbUBA1a and TbUBA1b than in hUBA1 (Boer and Bijlmakers, 2019). Furthermore, the rigidity that comes from P554 at the “far end” of this pocket may put constraints on the hUBA1 pocket that are not present in the *T. brucei* UBA1s (Figure 3). These important differences predict that parasite-selective inhibitors can be generated, for which the replacement of the trifluoromethyl group of TAK243 by bulkier groups may be a good starting point. Additionally, the TbUBA1 proteins can be used in high throughput enzymatic assays to screen for novel inhibitors.

Interestingly, the *T. cruzi* UBA1s, TcUBA1a and TcUBA1b, differ at the gatekeeper and the P554 equivalent as well (Figure 3). Like TbUBA1a, TcUBA1a has a serine at the gatekeeper position and like TbUBA1b, TcUBA1b has a threonine at this location. In *L. major*, LmUBA1a has a serine at the gatekeeper as well, but LmUBA1b contains the conserved alanine (Figure 3). On the other hand, LmUBA1a does not differ from hUBA1 at the P554 equivalent, whereas LmUBA1b does. Nevertheless, the LmUBA1a protein is also more than 100-fold less sensitive to TAK-243 compared to hUBA1, consistent with the importance of the difference at the gatekeeper residue (Boer and Bijlmakers, 2019). It remains to be determined whether the generation of a pan-trypanosomatid UBA1 inhibitor will be possible, or whether the differences between the trypanosomatids require inhibitors to be optimized for each species. It is also not yet known whether both UBA1s would need to be targeted in these trypanosomatids. The severity of the TbUBA1b knockdown argues that the sole inhibition of this protein would be sufficient for *T. brucei*, but such data are not available for *T. cruzi* and *Leishmania*. Importantly, a precedent for the targeting of UBA1 in parasites was recently set by the demonstration that TAK243 inhibits the growth and development of Plasmodium (Green et al., 2020). Treatment with TAK-243 was found to result in an absence of viable parasites released from red blood cells. An induced knockdown of UBA1 resulted in the same phenotype as TAK243 inhibition, also showing the power of targeting UBA1. The UBA1 of Plasmodium does not differ from hUBA1 at the gatekeeper position, and consistently, the recombinant protein was found to be inhibited by TAK-243 at similar IC50s as recombinant hUBA1.

### Targeting E1s of Ubiquitin-like Proteins

In addition to UBA1, the trypanosomatids contain genes for five other E1s, namely UBA2, UBA3, UBA5, MOCS3 and ATG7 (Boer and Bijlmakers, 2019). These are involved in the activation of ubiquitin-like proteins and work in cascades with E2 and E3 proteins similar to that of ubiquitination. UBA2 and UBA3 are the E1s for SUMO and Nedd8, respectively, the attachment of which also leads to changes in the activity of substrates. In contrast to UBA1, the E1s for SUMO and Nedd8 are heterodimers. The SUMO E1 is composed of the catalytic UBA2 in which the active adenylation domain, catalytic cysteine domain and the UFD are located, and the structurally important AOS1. A similar division of labor is found in the SUMO E3 that consists of the catalytic UBA3 and the structural APPBP1.

SUMOylation plays crucial roles in many cellular processes including gene expression, DNA repair, protein transport and cell cycle progression (Gareau and Lima, 2010). In accordance with this, sumoylation is essential for eukaryote survival. Trypanosomatids express only one SUMO protein, whereas there are four in humans. The RNAi knockdown of SUMO in *T. brucei* has been shown to be lethal in both the insect and blood stream form (Liao et al., 2010), showing that sumoylation plays an essential role in this parasite as well. Furthermore, data from a genome-wide RNAi screen in *T. brucei* show that knockdown of UBA2 and AOS1 reduce viability by 40 and 75%, respectively (Alsford et al., 2011).

The UBA2/AOS1 and the E2 dedicated to SUMOylation, Ubc9, of *T. brucei* have been purified and shown to have *in vitro* sumoylation activity (Ye et al., 2015). Some substrates have been identified in *T. brucei*, such as TbAUK1, an orthologue of Aurora B kinase that plays crucial roles in mitosis and cytokinesis. A mutation at the sumoylation site of this protein was shown to interfere with its function (Hu et al., 2014). Sumoylation has further been shown to positively regulate the expression of VSG, the variant surface protein of *T. brucei* that is key to its escape from the immune system (López-Farfán et al., 2014; Saura et al., 2019). Sumoylation of the transcription factor SNF2PH is essential for its presence at the VSG-ES (expression site) promoter and thus for transcription from this site. A proteomics approach with an affinity tagged SUMO identified 45 SUMOylated proteins in *T. brucei* with predominantly roles in the nucleus (Iribarren et al., 2015). In *T. cruzi*, a mass spectrometry-based approach detected more than 200 sumoylation substrates (Bayona et al., 2011). Parasite specific processes may also be regulated by SUMOylation in *T. cruzi* since this modification was not only detected in the nucleus but also on the flagellum. Here, the flagellar rod protein PFR1 was identified to be a SUMOylation substrate (Annoura et al., 2012).

Given the essential role of SUMOylation, inhibition of UBA2 is expected to be lethal and therefore provides another drug targeting possibility. Also for this protein there are indications that parasite selectivity may be achieved. ML-792 is an inhibitor that is selective for UBA2 and has the same mechanism of action as TAK-243 (He et al., 2017). This compound has been shown to be cytotoxic for human cells where it induces mitotic disruption. Mutations in UBA2 that cause a resistance to ML-792 have been identified and contained a substitution at S95 as well as M97, S95N/M97T. Significantly, the UBA2s of all three trypanosomatids do not contain a serine nor a methionine at the S95 and M97 equivalents, respectively. Moreover, the *T. brucei* and *Leishmania* UBA2s contain the resistance inducing asparagine at the corresponding position of S95. This indicates that also for this protein there are considerable differences in inhibitor binding properties between the human and the trypanosomatid form. This may represent another opportunity for the design of parasite-selective inhibitors with cytotoxic effects.

Finally, the knockdown of Nedd8 by RNAi has also been shown to be lethal in *T. brucei* (Liao et al., 2017). A major role of Nedd8 is the activation of cullin CRL-type ubiquitin ligases that play important roles in cell cycle progression. In agreement with this, Nedd8 deficient *T. brucei* showed a reduction in overall ubiquitination, mitotic defects and cell death. Moreover, six cullins were shown to be neddylated in *T brucei* and a further 70 substrates were identified by affinity purification followed by mass spectrometry. The deficiency in Nedd8 was further found to lead to a detachment of the flagella. Thus, given these essential roles of Nedd8 the trypansomatid UBA3 may be regarded as a drug target as well.

## Inhibition of the Proteasome

### Proteasome Inhibitors

Proteasome inhibitors have been used extensively in research and were instrumental in obtaining functional and mechanistic insights into proteasome activity (Fenteany et al., 1995; Lee and Goldberg, 1998; Groll and Potts, 2011). More recently they have entered the clinic as anti-cancer drugs. They have also been used in studies of trypanosomatids to determine the role of the proteasomes in the growth and differentiation of these parasites.

**FIGURE 4:**
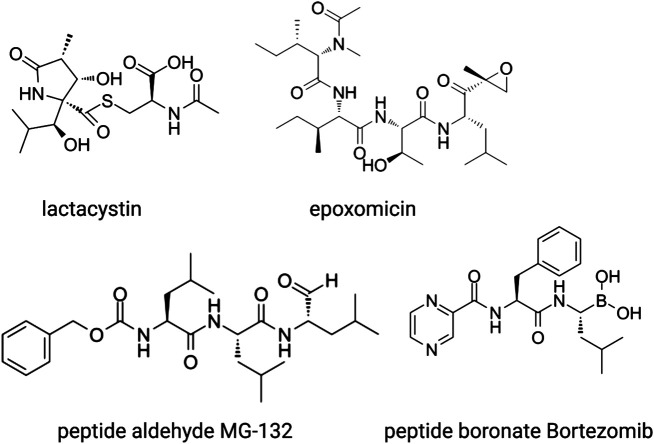
The main classes of proteosome inhibitors that target the proteolytic sites.

Proteasome inhibitors fall into several chemical categories and have been isolated from micro-organisms as well as rationally designed based on pre-existing knowledge of protease inhibition (Figure 4). One of the first molecules identified is lactacystin, a natural compound that was isolated from the soil-dwelling bacteria *Streptomyces* lactacystinaeus (Ōmura and Crump, 2019). Lactacystin is a lactam, or cyclic amide, that in aqueous solutions spontaneously converts into the active inhibitor clasto-lactacystin-β-lactone (Dick et al., 1996). This compound binds irreversibly to the catalytic amino-terminal threonine of the β5 and β2 subunit where it forms an ester adduct, and reversibly to that of β1, blocking all proteasome activity (Fenteany et al., 1995). Another naturally occurring γ-lactam-β-lactone is Salinosporamide A, a product of the marine bacteria Salinospora, which has a similar mode of action but is > 30x more potent than lactacystin (Feling et al., 2003).

Another natural compound, the linear peptide epoxyketone epoxomicin (Figure 4), was isolated from an Actinomycetes strain of bacteria. The selectivity of this inhibitor for the proteasome is even higher than that of lactacystin, which also inhibits the lysosomal protease Cathepsin A (Meng et al., 1999). Epoxomicin undergoes two subsequent rounds of nucleophilic attacks, first by the side chain hydroxyl of the N-terminal threonine and next by the free amine group, which results in an irreversible six-membered morpholino bond. Epoxomicin inhibits all three subunits, but by far the strongest effect is on β5. Other natural products with anti-proteasomal activity have been isolated from micro-organisms as well, such as the *ß*-lactone belactasin A, the aldehyde fellutamide B and the syrbactins that have yet another mode of action.

MG-132 was the first synthetic proteasome inhibitor to be generated (Lee and Goldberg, 1998). This compound belongs to the class of peptide aldehydes that were previously characterized as inhibitors of serine and cysteine proteases. The peptide part of MG-132 acts as a substrate analogue whereas the aldehyde is the pharmacophore that reacts reversibly with the hydroxyl group of the catalytic N-terminal threonine. MG-132 inhibits lysosomal cysteine proteases and calpains as well but at higher concentrations than required for proteasome inhibition. Peptide vinyl sulfones were designed as irreversible proteasome inhibitors and have been used as labelling probes to obtain insights into the proteolytic mechanisms and sequence preferences of the catalytic subunits as described above (Bogyo et al., 1997).

Since their first discovery, lactacystin and epoxomicin have been shown to inhibit cell cycle progression. Cancer cell lines and xenograft tumors in mice are considerably more sensitive to these inhibitors than non-transformed cells because of a higher dependence on protein quality control mechanisms that comes with their rapid proliferation rate and, in some hematological malignancies, because of ER stress coming from the production of large amounts of antibodies. Therefore, proteasome inhibitors were explored as anti-cancer drugs, which resulted in the development of bortezomib (Figure 4), a boronated dipeptide that is much more potent and selective than MG-132 and has good bioavailability (Adams, 2001). The boronate forms an adduct with the active site of the *ß* subunits, which is stabilized by a hydrogen bond between the free N-terminal amino group and one of the hydroxyl groups of the boronate. This explains its selectivity for N-terminal threonine proteases over other proteases. Bortezomib is now used in the clinic as first-line treatment for multiple myeloma and mantle cell lymphoma and is being considered for solid tumors. Novel proteasome inhibitors are being developed because of the occurrence of pre-existing or induced resistance to bortezomib and severe adverse effects. These include the epoxomicin-related carfilzomib, the naturally occurring *ß*-lactam marizomib (Salinosporamide), and the orally available boronated dipeptide ixazomib.

### Effects of Proteasome Inhibitors on Growth and Differentiation of Trypanosomatids

Proteasome inhibitors have also been shown to cause cell cycle arrest and cell death in trypanosomatids. Treatment of *T. brucei* blood stream form with lactacystin, for instance, causes an arrest in G1 and G2 and cell death by apoptosis after 24 h (Mutomba et al., 1997). Similar results were obtained with the MG-132 related tripeptide aldehyde inhibitor LLnV. Both lactacystin and LLnV were also toxic against the insect form of *T. brucei* although this arrest was at the G2/M phase and required 5–10 x higher concentrations and longer incubations than for the blood stream form. In contrast, the differentiation of the insect form into the blood stream form, a process that does not involve the crossing of cell cycle stage boundaries, was not inhibited by proteasome inhibitors (Mutomba and Wang, 1998).

However, lactacystin does inhibit the differentiation of *T. cruzi*. This parasite infects mammalian cells in the non-cycling trypomastigote form, which subsequently differentiates into amastigotes that replicate in the cytoplasm of the host cells. This differentiation can be mimicked under cell-free conditions, which was found to be strongly inhibited by lactacystin (González et al., 1996). It was further observed that the pretreatment of trypomastigotes with lactacystin did not prevent infection of cells, but resulted in a ∼75% reduction in intracellular parasites at 72 h after infection (González et al., 1996). This indicates that also intracellular differentiation was affected and perhaps amastigote proliferation as well. In the absence of inhibitor, amastigotes in the host cell undergo several days of cell division before differentiating into trypomastigotes that leave the cells. When established intracellular amastigote infections were treated with lactacystin, a ∼10-fold reduction in released trypomastigotes was observed together with a continued presence of intracellular amastigotes (González et al., 1996). This demonstrated that also the amastigote to trypomastigote differentiation is affected by proteasome inhibition. Another important process in the complicated life cycle of *T. cruzi* is metacyclogenesis, which takes place inside the triatominae and involves the differentiation of the non-infectious proliferating epimastigote into the infectious non-proliferating trypomastigote from. Lactacystin was found to inhibit both the growth of epimastigotes in culture (Cardoso et al., 2008) and metacyclogenesis (Cardoso et al., 2011). Similar results on differentiation were reported for *L. chagasi*, which alternates between the flagellated insect form, the promastigote, that infects human cells and the intracellular non-flagellated amastigote that replicates inside the phagolysosomes of the host cells. The proliferation of promastigotes in culture was inhibited by lactacystin but their ability to infect cells was not. However, pretreatment of promastigotes with lactacystin severely reduced the survival of the parasite inside host cells (Silva-Jardim et al., 2004).

Thus, although different effects on differentiation have been reported, proteasome inhibition is toxic for all three parasites.

### Trypanocidal Proteasome Inhibitors

The toxicity of proteasome inhibitors, together with the differences in substrate selection between trypanosomatid and mammalian proteasomes suggests that selective inhibition of parasite proteasomes can be achieved (Nkemgu-Njinkeng et al., 2002). In addition to the ones described above, a wide range of other proteasome inhibitors of different chemical classes have been shown to kill cultures of *T. brucei* blood stream forms, sometimes at lower EC50s than mammalian cells. These include various peptide aldehydes, epoxomicin and its derivatives, peptidyl vinyl sulfones, peptidyl vinyl esters, and several natural γ-lactam-β-lactones (Glenn et al., 2004; Steverding et al., 2005; Steverding et al., 2011a; Steverding et al., 2011b). Several studies have shown differences in the relative inhibition of β5 and β2 subunits between trypanosoma and human proteasomes, again indicative of the parasite proteasome possessing distinct catalytic activities (Nkemgu-Njinkeng et al., 2002; Wang et al., 2003; Zmuda et al., 2019). However, the design or selection of trypanosomatid-selective inhibitors based on these proteolytic differences has not yet been possible (Steverding et al., 2018). Nevertheless, in recent years impressive progress in the development of trypanosomatid-selective proteasome inhibitors with a promise for the clinic has been made. This progress has come from large phenotypic screens in which proteasome inhibitors with an unpredicted mode of action were identified.

**FIGURE 5:**
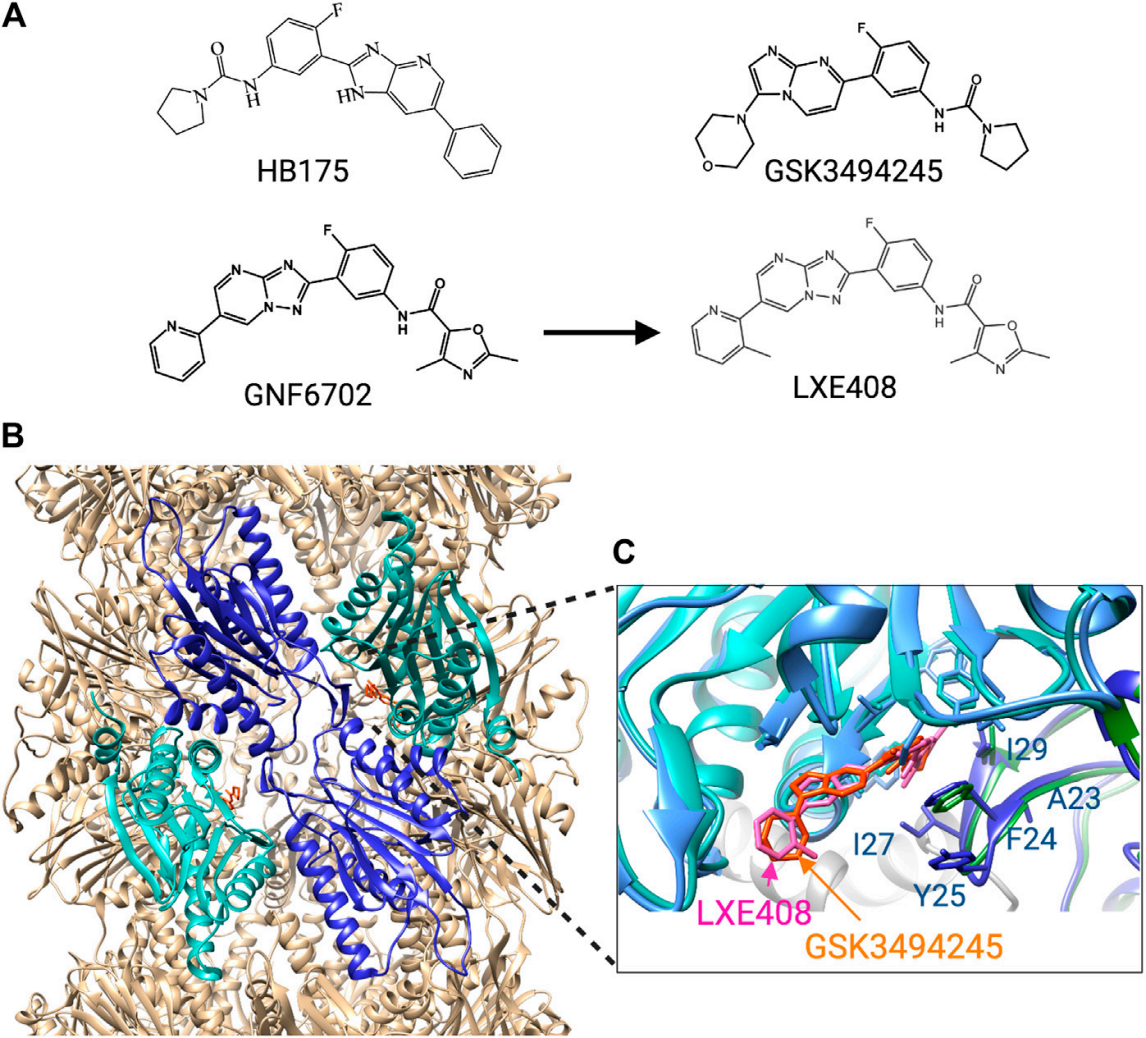
Trypanosomatid selective proteosome inhibitors. **(A)** structures of the inhibitors. **(B)** Cryo-EM structure of *L. tarantulae* proteosome complexed with GNF6702 (PDB 6QM7). The β5 subunits are in cyan, and the β4 subunits in blue. The inhibitor GNF6702 is in orange. **(C)** Detailed view of the inhibitor binding site located between the β4 and β5 subunits. The structure of the *L. tarantulae* proteosome with GNF6702 was overlayed with that of the *L. tarantulae* proteosome complexed with LXE408 (PDB6TCZ), showing the similar position of the two inhibitors. The residues of β4 that differ in human β4 are labeled.

The first breakthrough came from a screen of 3,000,000 compounds for cytotoxic effects on *T. brucei*, *T. cruzi* and *L. donovani* (Khare et al., 2016). A candidate molecule was selected based on potency, activity against all three parasites, and limited effects on mammalian cells. This molecule was modified to obtain GNF6702 (Figure 5) with better bioavailability and enhanced potency against intramacrophage *Leishmania*. In mouse models, GNF6702 given via oral gavage was found to be at least as effective as existing drugs against visceral leishmaniasis (*L. donovani* infection), cutaneous leishmaniasis (*L.major* infection), Chagas’ disease (chronic *T. cruzi* infection) and stage II HAT (meningoencephalic *T. brucei* infection) (Khare et al., 2016). GNF6702 reduced the number of *L. donovani* by 90% in all five treated mice, cleared *T. cruzi* from all but one out of eight mice, and cured *T. brucei* infection up to 94 days of monitoring in all six mice. In the latter case the compound had to be given at a 10-fold higher concentration (100 mg/kg) because of poor brain accessibility.

The mechanism of GNF6702 was determined by evolving drug-resistant *T. cruzi* strains, which identified the non-proteolytic β4 subunit of the proteasome as a target (Khare et al., 2016). Overexpression of a β4 protein carrying the mutations detected in the resistant strains, F24L and I29M, rendered *T. cruzi* and *T. brucei* resistant to GNF6702 but not to bortezomib or MG-132. This is consistent with an alternative mode of inhibition that does not affect the active site of the catalytic proteasome subunits. GNF6702 was further shown to inhibit the chymotrypsin-like activity of the β5 subunit, but not the trypsin- and caspase-like activity of the other proteolytic subunits. This, together with the location of the resistance-inducing mutations suggested an allosteric inhibitory mechanism in which the molecule binds in a pocket between the β4 and β5 subunit.

Insights into such an allosteric inhibition came from the synthesis of GSK3494245 (Figure 5), based on a hit from another phenotypic screen (Wyllie et al., 2019). GSK3494245 reduces parasite load by 95% after 10 days in a mouse model of visceral leishmaniasis, similar to treatment with the current drug miltefosine. GSK3494245 is an imidazopyrimidine and thus related in structure to the triazolopyrimidine GN6702. Therefore, its effect on the proteasome was investigated which demonstrated that like GN6702, GSK3494245 inhibits the chymotrypsin- but not the trypsin- and caspase-like activity. Importantly, GSK3494245 was > 100-fold less active against human proteasomes. Consistent with a mechanism that involves the proteasome, *Leishmania* strains with evolved GSK3494245 resistance were found to have mutations at T30 of β4 and G197 of β5, both of which are located at the β4 - β5 interface (Wyllie et al., 2019).

The binding mode of GSK3494245 was conclusively determined by cryo-EM using *L. tarantulae* proteasomes (Wyllie et al., 2019). This showed that GSK3494245 binds between the β4 and the β5 subunit but, unexpectedly, protrudes mostly into the β5 subunit (Figure 5). Only the pyrrolidine carboxamide group binds in a narrow hydrophobic pocket of β4 where it makes contact with six β4 residues in addition to three from β5. Significantly, the majority of the human β4 residues at equivalent positions (Figure 5) are different and the corresponding cavity in the human protein is more open, shallow and solvent exposed. This provides a plausible explanation for the lack of GSK3494245 activity against human proteasomes. The G197 residue of β5 identified in GSK3494245-resistant *Leishmania*, makes direct contact with the inhibitor, whereas the similarly identified T30 of β4 is likely to be important for access to the β4 pocket. Also β4 residues F24 and I29 that were mutated in the GNF6702-resistant cells, make contact with GSK3494245 (Figure 5).

Treatment of *L. donovani* cultures with GSK3494245 resulted in an accumulation of ubiquitinated proteins, swelling of the parasite and the presence of vesicles indicative of proteotoxic stress, and an arrest at G2/M, all consistent with a targeting of the proteasome (Wyllie et al., 2019).

Yet another large phenotypic screen identified an oxalopyridine with cytocidal effects on *T. brucei*, which led to the generation of an imidazopyridine referred to as compound 64 (Tatipaka et al., 2014), later called HB175 (Figure 5). This molecule was shown to cure acute hemolymphatic HAT in a mouse model. Based on the resistance of a *T. brucei* strain with a β4 mutation at F24 to HB175, it was concluded that this molecule is also a proteasome inhibitor (Nagendar et al., 2018). A comparative study analyzed the effects of HB175 and 16 new imidazopyridines alongside GNF6702 and 12 new triazolopyrimidines, which showed that compounds of both classes kill *T. brucei* and intracellular *T. cruzi* at EC50s below 100 nM (Nagendar et al., 2018). While the imidazopyridines were on average slightly more potent, they also showed greater toxicity against a human lymphocytic cell line. Another more favourable characteristic of the triazolopyrimidines was their greater stability in the presence of mouse liver microsomes, which corresponded to a higher blood exposure in mice. The brain penetration of the triazolopyrimidines was also higher than that of the imidazopyridines although this was still not higher than 12% for the triazolopyrimidine GNF6702. In this study, the triazolopyrimidines were therefore only tested against *T. cruzi* and not against *T. brucei* infections. GNF6702 was found to be more potent than two of the novel triazolopyrimidines in a mouse model of acute *T. cruzi* infection, in which mice are infected with a parasite dose that kills 95% within 20 days of infection. Treatment of mice on days 7–11 after infection with GNF6702 resulted in the absence of detectable parasites for 42 days, whereas this was only 13 days for the current drug benznidazole.

In another study, GNF6702 was investigated against *T. brucei* infection together with two related triazolopyrimidines, GNF3849 and NITD689 (Rao et al., 2020). All three compounds were able to cure hemolymphatic HAT when given orally three days after infection for four days and mice stayed free of *T. brucei* up to 30 days of monitoring. In a stage II HAT model, mice treated with GNF6702 became parasite free and remained so for 94 days at a dose that was ∼3x lower than in the first report on this compound (30 mg/kg instead of 100 mg/kg). Of the three compounds, GNF6702 had the most desirable characteristics since both GNF3849 and NITD689 showed limited *in vivo* availability, GNF3849 because of high protein binding properties and NITD689 because of low stability.

Although GNF6702 showed promising results, its solubility was too limited to make an oral formulation possible (Nagle et al., 2020). The insolubility was predicted to come from the planar shape of the molecule that leads to a crystallizing tendency. To force the molecule out of plane, groups were attached to the pyridine ring which resulted in a more soluble molecule, LXE408 (Figue 3), that retained good anti-proteasomal activity. Crucially, oral administration of LXE408 had greater efficacy in a visceral leishmaniasis model than miltefosine, the only oral medication available, and was as effective as the most potent drug amphotericin B in cutaneous leishmaniasis (Nagle et al., 2020).

The cryo-EM structure of LXE408 bound to *L. tarantulae* proteasomes showed that this molecule binds in a way analogous to that of GSK3494245 (Nagle et al., 2020) (Figure 5). Thus, most of the molecule protrudes into the β5 subunit and only the dimethyl-oxazole packs against β4 residues that include F24 and I29. A structure of proteasomes with both LXE408 and bortezomib showed that bortezomib adopts a novel conformation in the presence of LXE408, which explains why the two inhibitors do not compete although their binding sites partially overlap.

LXE408 has a desirable safety profile and is now being tested in phase I clinical trials (Nagle et al., 2020). GSK3494245 also showed reasonable bioavailability and a good safety margin in rat and is being tested in phase I trials as well (Wyllie et al., 2019; Zmuda et al., 2019).

Thus, there are now two candidates of proteasome inhibitors with good efficacy, desirable safety profiles and the promise of short oral treatments. However, more molecules are needed to increase the pipeline given the low success rates of clinical trials. To identify these, target-based screens will also play important roles. To this end, a luminescence-based assay for chymotrypsin-like activity was optimized for high throughput screening with *T. cruzi* proteasomes (Zmuda et al., 2019). The assay was validated with peptide aldehyde (MG132, MG115), peptide boronate (bortezomib, ixazomib) and peptide epoxyketone inhibitors (epoxomicin, oprozomib). Following this, a screen with 18,039 compounds was performed that after a counter screen for technology interfering compounds resulted in 39 hits. Details of the identified molecules have not yet been reported. In addition, a novel tool for the selective screening of proteasome inhibitors in live *T. brucei* has been developed. This involves a modified *T. brucei* strain that expresses a GFP-tagged mutant ubiquitin molecule that cannot be hydrolyzed by deubiquitinating enzymes (Moura et al., 2018). Proof of principle experiments showed that treatment with lactacystin and MG-132 led to an accumulation of GFP-ubiquitin.

## Concluding Remarks

The ubiquitin-proteasome system represents a good theoretical drug target in trypansomatids because of its essential functions. However, there is now also substantial data demonstrating that selectively targeting this system in these parasites is indeed feasible. This is clearly proven by the development of trypanosomatid selective proteasome inhibitors that target this protein complex in a way not previously envisioned. The current inhibitors are promising, but general success rates of clinical trials are low and so further drug candidates are required. Moreover, there are additional challenges unique for these diseases such as the need for cheap, orally available compounds that can cure during a short treatment. Challenging cellular and tissue distribution criteria need to be met as well, such as the delivery to phagolysosomes for leishmaniasis and the ability to cross the blood-brain barrier for stage II HAT. For these reasons, it will be valuable to continue drug discovery programs including target-based high throughput screening of proteasomes either in purified form or in live trypanosomas modified for this purpose. Additionally, it will be important to explore the alternative, complementary strategy of inhibiting UBA1 enzymes. Trypanosomatid-selective inhibitors of this protein are not yet available, but there is a good basis to predict that these can be obtained. Using TAK-243 as a starting point may be an effective way to achieve this, but also high throughput assays can be used to enquire additional chemical space. Since the consequences of ubiquitination are more widespread than that of proteasome inhibition, the inhibition of UBA1 may have more potent effects than the inhibition of the proteasome. It can also be envisaged that the complementary effects of proteasome and UBA1 inhibitors may be combined in drug treatment. Finally, further promise lies in the targeting of UBA2 and UBA3 that stand at the helm of essential processes as well and have not been explored for this purpose yet.
